# Leaf nonstructural carbohydrate concentrations of understory woody species regulated by soil phosphorus availability in a tropical forest

**DOI:** 10.1002/ece3.6549

**Published:** 2020-07-14

**Authors:** Qifeng Mo, Yiqun Chen, Shiqin Yu, Yingxu Fan, Zhongtong Peng, Wenjuan Wang, Zhi’an Li, Faming Wang

**Affiliations:** ^1^ Guangdong Key Laboratory for Innovative Development and Utilization of Forest Plant Germplasm College of Forestry and Landscape Architecture South China Agricultural University Guangzhou China; ^2^ Key Laboratory of Vegetation Restoration and Management of Degraded Ecosystems and Xiaoliang Research Station of Tropical Coastal Ecosystems South China Botanical Garden Chinese Academy of Sciences Guangzhou China

**Keywords:** N and P addition, P limiting, soluble sugar, starch, tropical forest

## Abstract

Leaf soluble sugars and starch are important components of nonstructural carbohydrates (NSCs), which are crucial for plant growth, development, and reproduction. Although there is a large body of research focusing on the regulation of plant NSC (soluble sugars and starch) concentrations, the response of foliar NSC concentrations to continuous nitrogen (N) and phosphorus (P) addition is still unclear, especially in tropical forests. Here, we used a long‐term manipulative field experiment to investigate the response of leaf NSC concentrations to continuous N and P addition (3‐, 5‐, and 8‐year fertilization) in a tropical forest in southern China. We found significant species‐specific variation in leaf NSC concentrations in this tropical forest. Phosphorus addition dramatically decreased both leaf soluble sugar and starch concentrations, while N addition had no significant effects on leaf soluble sugar and starch concentrations. These results suggest that, in plants growing in P‐limiting tropical soil, leaf NSC concentrations are regulated by soil P availability rather than N availability. Moreover, the negative relationships between NSC concentrations and leaf mass per area (LMA) revealed that NSCs could supply excess carbon (C) for leaf expansion under P addition. This was further supported by the increased structural P fraction after P fertilization in our previous study at the same site. We conclude that soil P availability strongly regulates leaf starch and soluble sugar concentrations in the tropical tree species included in this study. The response of leaf NSC concentrations to long‐term N and P addition can reflect the close relationships between plant C dynamics and soil nutrient availability in tropical forests. Maintaining relatively higher leaf NSC concentrations in tropical plants can be a potential mechanism for adapting to P‐deficient conditions.

## Graphical abstract

Maintaining relatively higher leaf NSCs in tropical plants can be a potential mechanism adapting to the P‐deficient conditions. The response of leaf NSCs pool to long‐term N and P addition can reflect the tight relationships between plan C dynamic and soil nutrient availability in tropical forests.

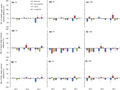

## 1 INTRODUCTION

In plants, carbohydrates, which are classified as structural carbohydrates (SCs, including lignin and cellulose) and nonstructural carbohydrates (NSCs, including soluble sugar, sucrose, fructose, and starch), are of great importance to energy sources and physiologicalbolism in plant life history (Dietze et al., 2014; Ögren, 2010). SCs are generally used for constructing plant tissue, and NSCs mainly offer carbon (C) and energy for plant growth, respiration, and production (Dietze et al., 2014; Würth, Peláez‐Riedl, Wright, & Körner, 2005).

Nonstructural carbohydrates are referred to as reservoir pools, and have protective functions, including osmotically active compounds, chemical chaperones, and reactive oxygen species (ROS) scavengers (Ivanov et al., 2019; Newell, Mulkey, & Wright, 2002). Generally, NSCs only account for approximately 10% of plant biomass, but their concentrations in leaves are higher than those in roots and stems under natural conditions, indicating the vital role of plant leaves in regulating the C balance between uptake and consumption (Martínez‐Vilalta et al., 2016). The concentrations of NSCs can mirror the capacity of plant adaption to the various environmental conditions (Hoch, Richter, & Korner, 2003; Richardson et al., 2015). Under exposure to various global environmental changes, such as warming, CO_2_ enrichment, ozone destruction, drought, and N deposition, plant survival, resistance ability, growth rate, and productivity are primarily determined by carbohydrate dynamics (Dietze et al., 2014; Martínez‐Vilalta et al., 2016).

It is widely believed that N and P are two essential nutrients for plant photosynthetic C assimilation, and they also limit the net primary productivity (NPP) in terrestrial ecosystems (Herbert & Fownes, 1995; Vitousek & Howarth, 1991). Traditionally, N availability constrains plant productivity by limiting leaf initiation and expansion (Vos & Biemond, 1992), while P availability mainly determines leaf biochemical processes such as energy exchange and nucleic acid synthesis in plant cells (Warren, 2011). In the herbaceous plant yellow bluestem (*Bothriochloa ischaemum*), the soluble sugar concentrations were reduced while the starch and total NSC concentrations were increased by N addition (Xiao, Liu, Li, & Xue, 2017). In another study, both above‐ and below‐ground NSC (sugars and starch) concentrations in yellow bluestems were significantly increased by N addition (Ai, Xue, Wang, & Liu, 2017). Nonetheless, both N and P addition decreased the concentrations of leaf soluble sugars and starch in two species of grass and forbs in an Inner Mongolian semi‐arid grassland community (Wang, Xu, et al., 2017). Al‐Hamdani and Sirna (2008) also reported that the starch and total NSC accumulation in *Salvinia minima* were significantly lower under N or P addition. However, previous studies on different plant species (which mainly include herbaceous species) have found different results regarding the plant's responses to external N and/or P addition.

In tropical forests, P is an important limiting factor for plant growth and productivity (Vitousek, Porder, Houlton, & Chadwick, 2010), as soil P availability generally declines with bedrock weathering and soil age (Walker & Syers, 1976). Therefore, soil P availability in tropical forests may drive leaf NSC dynamics, which could reflect carbohydrate dynamics (C assimilation by photosynthesis and consumption by respiration). Although it is widely recognized that P addition could greatly increase leaf P concentrations in the tropics (Mayor, Wright, Turner, & Austin, 2014; Schreeg, Santiago, Wright, & Turner, 2014; Wright et al., 2018), very few studies have investigated how increased leaf P concentrations affect the NSC dynamics in tropical forests.

A recent study reported that leaves tended to optimally allocate different functional P fractions (structural P, metabolic P, nucleic acid P, and residual P) to simultaneously accomplish a series of physical processes (photosynthesis) in P‐limiting tropical forests (Mo et al., 2019). Given that leaf C assimilation is closely related to N and P supply (Kroth, 2015), the response of NSC to long‐term N and P addition is relatively fundamental for understanding the mechanisms and relationships of leaf C assimilation and P allocation in tropical forests.

Fertilization experiments involving addition of external N and P on reforestation from degraded sites have been conducted at many locations globally (Ceccon, Huante, & Campo, 2003; Li et al., 2015; Mayor et al., 2014; Schreeg et al., 2014; Tanner, Kapos, & Franco, 1992). These experiments could be efficient ways to evaluate the effects of P limitation on key biological process (Ågren, Wetterstedt, & Billberger, 2012; Tessa, Hättenschwiler, Treseder, Lehmann, & Rillig, 2018). In tropical China, N deposition is projected to continually increase in the future (Liu et al., 2011). The increased atmospheric N input may provide higher active N for plant growth in forest ecosystems (Lu, Mo, & Dong, 2008), which also aggravates the imbalance of soil N:P ratios in tropical forests (Du et al., 2016). According to our previous results, the plant growth in this studied secondary tropical forest has been proven to be primarily limited by soil P availability (Mo et al., 2015, 2019). Here, we employed a long‐term manipulative field experiment to test the response of leaf NSCs to N and/or P addition in a secondary tropical forest in southern China. We tried to answer the following questions: how do leaf NSC (soluble sugars and starch) concentrations respond to continuous N deposition and P limitation? How does N and P addition regulate leaf NSC concentrations in this tropical forest? We hypothesized that: (1) the concentrations of leaf NSCs (soluble sugars and starch) would be primarily regulated by P availability rather than N availability due to the long‐term P deficiency in this tropical forest, and (2) the NSC concentrations would be reduced and transformed into leaf biomass along with the increase in leaf structural P fraction under P addition (Mo et al., 2019).

## 2 METHODS

### 2.1 Site description

This study was conducted at the Xiaoliang Research Station for Tropical Coastal Ecosystems of the Chinese Academy of Sciences (Xiaoliang Station, 21°27′N, 110°54′E). Xiaoliang Station is located in the southwest of Guangdong Province, China, and has a tropical monsoon climate. In the studied region, the mean annual temperature is 23ºC, and the mean annual precipitation ranges from 1,400 to 1,700 mm. There is a clear seasonal variation, with the wet season lasting from April to October, and the dry season lasting from November to March. The soil is classified as a latosol developed from granite (Wang et al., 2014). It is estimated that the annual wet N deposition was approximately 40 kg N/ha in 2012 in this region (Mo et al., 2015), and the forest is generally regarded as a P‐limited ecosystem (Mo et al., 2019).

The study site was located approximately 5 km from the coast in a secondary broad‐leaf mixed forest on coastal land with a very small slope, which was. The forest was restored from a Queensland peppermint (*Eucalyptus exserta*) plantation by introducing 312 plant species between 1964 and 1975. Thereafter, natural colonization during succession displaced most of the planted tree species, and the area developed as a relatively typical secondary evergreen broad‐leaf mixed forest, with a high biodiversity and a complex community that was similar to a natural forest (Chen et al., 2016). The most common tree species in the study site are as follows: *Castanopsis fissa*, *Cinnamomum camphora*, *Carallia brachiata*, *Aphanamixis polystachya*, *Ternstroemia pseudoverticillata*, *Acacia auriculaiformis*, *Cassia siamea*, *Albizia procera*, *A. odoratissima*, *Leucaena leucocephala*, *Aquilaria sinensis*, *Chakrasia tabularis*, *Syzygium levinei*, *Schefflera heptaphylla*, *S. hancei*, *S. bullockii*, *Uvaria macrophylla*, *Schefflera octophylla*, *Psychotria rubra*, *and Aporusa dioica* (Li et al., 2015; Wang, Ding, et al., 2017).

### 2.2 Experimental design

A randomized block design experiment with N and/or P fertilization was established in the secondary tropical forest in September 2009 (Chen et al., 2016; Zhao et al., 2014). In each block, four plots (10 m × 10 m) were included, with five replicate blocks in total, and the adjacent blocks were separated by a 50‐m buffer region. The four treatments, including N addition (+N), P addition (+P), N and P addition (+NP), and a control (CK, no addition of mineral nutrients), were assigned randomly to the four plots within each block. The edges of each plot were trenched to a depth of 20‐cm, put by a PVC broad and surrounded by a 2 m wide buffer. Because a large number of fine roots were distributed in surface soils, the trenches largely inhibited the transfer of nutrients among different treatments, as evidenced by clear differences in extractable soil P between fertilized and unfertilized treatments after six years of fertilization in 2015 (Mo et al., 2019). In this study, the soil available P concentrations were significantly different between unfertilized and fertilized treatments, as described in our previous studies (Li et al., 2015; Mo et al., 2019).

Fertilizers were regularly applied bi‐monthly from 2009 to 2017 to achieve the total amounts of N and P equivalent to 100 kg ha^−1^ yr^−1^. Every fertilizer application consisted of quantitative fertilizer (NH_4_NO_3_ and/or Na_2_HPO_4_) were dissolved in 30 L of groundwater, which was then applied to the corresponding plots uniformly using a backpack sprayer near the soil surface. Thirty liters of groundwater was also applied to control plots (Li et al., 2015; Wang et al., 2014). It is estimated that the amount of added water in each plot was equivalent to 0.08% and 0.35% of rainfall inputs in the wet and dry seasons, respectively, so that the effects of added water on soil moisture could be ignored (Mo et al., 2015).

### 2.3 Sampling and measurements

Based on previous vegetation surveys at the study site, we selected four native woody tree species that occurred sufficiently frequently in plots in the four treatments, including *S. bullockii*, *U. macrophylla*, *S. octophylla*, and *P. rubra* (Table 1), all of which are perennial shrubs or small trees with shade tolerance and C3 carbon fixation, or evergreen tree species. Leaf samples were collected in June or July of 2012, 2015, and 2017 (continuous yearly sampling would be harmful to the sampled shrub individuals because a relatively large leaf amount was sampled in every sampling event). Briefly, we randomly selected three to five individuals of each species in each treatment, and then sampled fully expanded and healthy mature leaves. After labeling the samples in Ziploc bags, all leaf samples were immediately transferred back to the laboratory. Each leaf sample was washed and air‐dried, then placed in an oven at 105℃ for 30 min and maintained at 65℃ for 72 hr to a constant weight. Subsequently, leaf samples were ground and sieved using 0.15 mm sieves, after removing petioles and main veins, and leaf P concentrations were measured spectrophotometrically after digestion with sulfuric acid (H_2_SO_4_). Leaf N concentrations were determined using the Kjeldahl method (Wang et al., 2013).

**TABLE 1:** The characteristics of selected four woody tree species in unfertilized plots of tropical forest in 2011, South China

Family	Species	Density (individuals/hm^2^)	Ground Diameter (cm)	Height (m)	Canopy (cm × cm)	Life history
Myrtaceae	*Syzygium bullockii*	1,400	1.61	0.92	27.33 × 40.33	Shrub, native, evergreen
Annonaceae	*Uvaria microcarpa*	300	2.13	1.04	56.25 × 81.07	Shrub, native, evergreen
Rubiaceae	*Psychotria rubra*	200	0.78	0.62	32.50 × 40.00	Shrub, native, evergreen
Araliaceae	Schefflera octophyll^*^	5,900	2.36	1.08	43.38 × 59.29	Shrub, native, evergreen

The leaf mass per area (LMA) was measured in 2015 using a portable leaf area meter (LI‐3000A, LI‐COR Biosciences). Photosynthetic nutrient use efficiency for N (PNUE) and P (PPUE) was defined as the rate of net photosynthesis per unit N or P expressed on a leaf dry mass basis. Leaf P was partitioned into four fractions: structural P, metabolic P (including P*_i_*), nucleic acid P, and residual P, using sequential extraction (following Kedrowski (1983) with modifications by Hidaka and Kitayama (2011)). Details on how these measurements were conducted can be found in Mo et al. (2019).

The standard anthrone colorimetric method was employed to coarsely measure the concentrations of leaf NSCs, for the rapid analysis of large samples (Buysse & Merckx, 1993; Dubois, Gilles, Hamilton, Rebers, & Smith, 1956; Hewitt, 1958; Li, He, Yu, Wang, & Sun, 2016). For the determination of soluble sugar concentration (total sugar, sucrose, and fructose), 0.1 g of ground leaf sample was placed into a 50 ml tube and mixed with 10 ml 80% (v/v) alcohol, extracted in 90℃ water for 10 min, three times. All the extracted solutions were transferred into 50 ml flasks, and the final volume was adjusted to 50 ml for the measurement of soluble sugar via the standard anthrone colorimetric method. The residue after three extractions in the 50 ml tube was treated with 30% (V/V) perchloric acid (HClO_4_) for 12 hr and then extracted in 80℃ water in 10 min. After that, the extracted residue was cooled down and filtered, with the final volume adjusted to 50 ml in a flask for the determination of starch. Finally, the sugar and starch concentrations in all the extracted solutions were measured from the absorbance of anthrone colorimetric at 620 nm using a UV‐vis spectrophotometer. The soluble sugars and starch concentrations were calculated per dry matter of leaves (mg/g) (Li, He, et al., 2016; Xie, Yu, & Cheng, 2018). In this study, the NSC concentrations can be defined as the sum of the soluble sugar concentrations and starch concentrations (Hoch et al., 2003).

### 2.4 Data analysis

**FIGURE 1:**
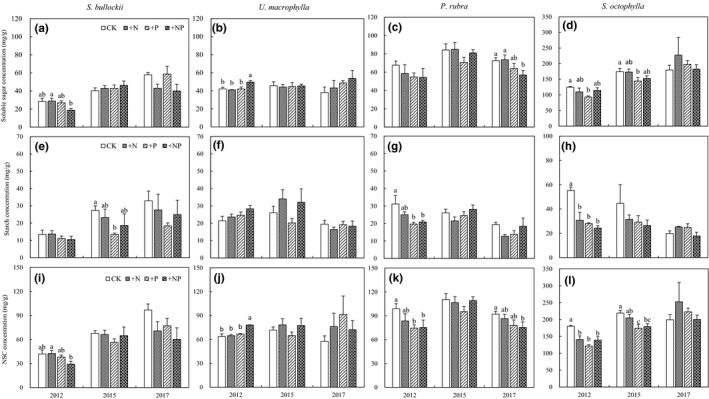
Foliar soluble sugar, starch, and NSC concentrations of four woody species sampled in 2012, 2015, and 2017 in a secondary tropical forest of southern China. The lowercase indicated the significant difference among four treatments with each species and each year

As our experiment used a randomized block design, for each species, we used species‐specific linear mixed model analysis to examine the effects of N, P, and N × P on the leaf soluble sugar and starch concentrations, and N, P, and N:P ratios for each species in 2012, 2015 and 2017. Given the variation among species, the linear mixed models were used to test the difference on the soluble sugar and starch, N, P concentrations and N:P ratios. In these models, species (S), N addition (N), and P addition (P) were considered as the fixed effects, and block, nested by sampling year, was included in the models as a random factor. Relative effects (RE) were quantified by the ratio of the variable in the experimental group (+N, +P, +NP) to the control group (CK), minus one (In Figure 1). All data analyses were performed in Excel 2013 and IBM SPSS Statistics 19.0. Results are reported as significant when *p* < .05.

## 3 RESULTS

### 3.1 Interspecific variation in leaf NSC

In this study, there were significant species variations in leaf soluble sugar, starch, and NSC concentrations (*p* < .001 for all, Table 2). In the CK treatment, the leaf soluble sugar concentration of *S. octophylla* was generally higher than that of the other three species, with concentrations of 124.54, 174.28, and 179.58 mg/g, in 2012, 2015, and 2017, respectively. In addition, the starch concentration of *S. octophylla* in the CK treatment was approximately twice as high as the other three species (Figure 1e–h). The NSC concentrations of *S. octophylla* in the CK plot were generally higher than those of the other three species (Figure 1i‐1), with concentrations of 179.81, 219.00, and 199.44 mg/g, in 2012, 2015, and 2017, respectively.

**TABLE 2:** Significance (*p*‐values) of terms from linear mixed model analysis for foliar soluble sugar, starch, NSC, soluble sugar/starch ratios of four woody species in a tropical forest following the fertilization with nitrogen (N) and phosphorus (P), where Y is year; S is species; N is N addition; and P is P addition

Variable	Soluble sugar concentration	Starch concentration	NSC concentration	Soluble sugar/Starch
Y	< **0.001**	**<0.001**	**<0.001**	**<0.001**
S	**<0.001**	**<0.001**	**<0.001**	**<0.001**
N	0.719	0.219	0.746	0.368
P	**0.016**	**0.001**	**<0.001**	0.193
S × N	0.259	0.068	0.355	0.666
S × P	**0.013**	**0.033**	**0.002**	0.473
N × P	0.866	**0.030**	0.320	0.779
S × N × P	0.836	0.866	0.836	0.179

Note

The bold value indicates significant effect of corresponding variables.

### 3.2 Leaf NSC concentration in response to N and P addition

**FIGURE 2:**
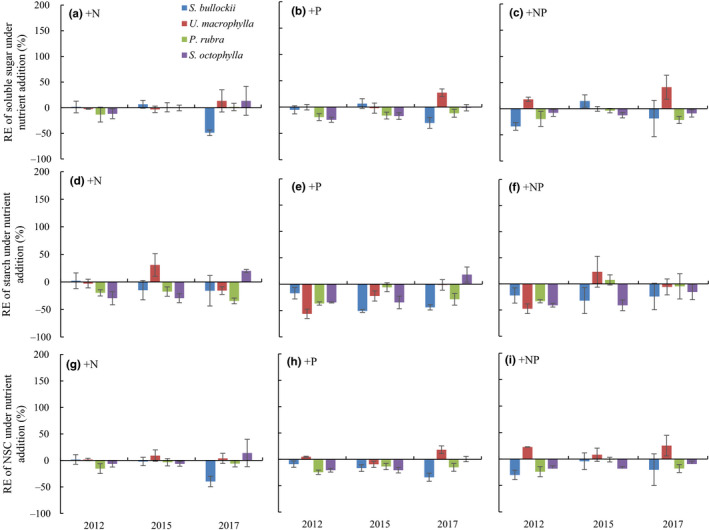
Relative effects (RE) of leaf soluble sugar (a, b, c), starch (d, e, f) and NSC (g, h, i) concentrations under + N, +P, and + NP treatments of four woody tree species in a tropical forest of southern China. Error bars were standard deviations. RE was quantified by the ratio of the variable in the experimental group (+N, +P, +NP) to the control group (CK) minus one

Neither N addition nor N × P interactions had significant effects on the total leaf NSC concentrations (*p* = .746 and *p* = .320, respectively), while P addition significantly affected leaf NSC concentrations (*p* < .001). P addition significantly influenced the NSC concentrations of *U. macrophylla*, *S. octophylla*, and *P. rubra* in 2012, those of *S. octophylla* in 2015, and those of *P. rubra* in 2017. Overall, +P reduced the NSC concentrations by 18.14% (except for *U. macrophylla*), 15.13%, and 24.68% (except for *U. macrophylla* and *S. octophylla*), in 2012, 2015, and 2017, respectively (Figure 2).

In this tropical forest, neither N addition nor N × P significantly affected leaf soluble sugar concentrations (*p* = .719 and *p* = .866, respectively, Table 2). However, the leaf soluble sugar concentrations were significantly changed by P addition (*p* = .016). Overall, +P decreased the leaf soluble sugar concentrations by an average of 12.78% across the three sampled years (except for *S. bullockii* in 2012 and *U. macrophylla*in 2017, Figure 2). Neither N nor P addition had significant effects on leaf soluble sugar concentrations in any of the species in 2012 (Table S1). However, there was a significant reduction in the soluble sugar concentrations of *S. octophylla* in 2015 and those of *P. rubra* in 2017 under P addition (Table S1).

Nitrogen addition did not change the leaf starch concentrations of the sample species (Table 2). However, both P addition and N × P interactions significantly affected starch concentrations (*p* = .001 and *p* = .030, respectively). Overall, +P reduced leaf starch concentrations by 38.10% (except for *S. octophylla* in 2017, Figure 2), +NP also decreased the starch concentrations by 28.28% (except for *U. macrophylla* and *P. rubra* in 2015, Figure 2). Additionally, P addition also had significant effects on the leaf starch concentrations of *P. rubra* in 2012, and those of *S. bullockii* in 2015 (Table S1).

Although the ratios of soluble sugar to starch showed significant interspecific variation (*p* < .001), this ratio was generally not affected by N, P, nor N × P interaction at the intraspecies level in this study (Table S1).

### 3.3 Relationships between leaf NSC and other traits

Leaf soluble sugar and total NSC concentrations, and the ratios of soluble sugar to starch were significantly negatively related to the LMA (Table 3). Leaf starch concentrations were significantly positively correlated with photosynthesis (*p* < .05). In addition, both the soluble sugar and NSC concentrations were significantly related to the leaf N concentrations (*p* < .05). Although there was no clear relationship between NSC (soluble sugar and starch) and leaf P concentrations, the structural P concentration had significantly positive correlations, while residual P concentration had significantly negative correlations, with soluble sugar and NSC concentrations, and the ratios of soluble sugar to starch (*p* < .05, Table 3).

**TABLE 3:** Correlation coefficients of leaf N:P stoichiometry and NSC variables of four species in a tropical forest in 2015, where NSC is nonstructural carbohydrates, LMA is leaf mass per unit area; [N] is N concentrations, [P] is P concentrations, PNUE is photosynthetic N‐use efficiency, and PPUE is photosynthetic P‐use efficiency

Variables	Soluble sugar	Starch	NSC	Soluble sugar/Starch
Photosynthesis	0.100	0.216*	0.144	0.073
Leaf area	0.538**	0.135	0.524**	0.394**
LMA	−0.456**	−0.121	−0.446**	−0.366**
[N]	0.249*	0.145	0.262*	0.100
[P]	0.092	0.015	0.088	0.064
N:P ratios	0.057	0.115	0.080	−0.030
PNUE	−0.005	0.110	0.022	0.046
PPUE	0.042	0.224*	0.092	0.013
Structural P	0.469**	0.165	0.468**	0.339**
Metabolic P	0.094	0.022	0.091	0.069
Nucleic acid P	−0.210	−0.153	−0.229*	−0.070
Residual P	−0.320**	−0.110	−0.319**	−0.278**

Note

* indicates significance at the 0.05 level.

** indicates signficance at the 0.01 level.

## 4 DISCUSSION

### 4.1 Effects of N and P addition on leaf NSC

Compared with starch, soluble sugars are relatively more mobilizable, and participate in many physiological and metabolic activities, including the regulation of cell osmotic pressure and transport systems involved in plant growth (Hoch et al., 2003; Millard & Grelet, 2010). However, starch is supposed to be the storage compound, and its pool could be depleted and changeable under the adverse environmental conditions (Ivanov et al., 2019; Martínez‐Vilalta et al., 2016). Exogenous N fertilization has been proven to increase the plant growth rates and productivity in global tropical forests (Pasquini & Santiago, 2012; Selene & Jürgen, 2018), and also alters the NSC allocation pattern in tropical trees (Burslem, Grubb, & Turner, 1996). Surprisingly, we found that continuous N fertilization did not change the leaf NSC concentrations (Table 2). This may be due to the relatively higher leaf N contents and “N‐saturation” in the studied forest (Lu et al., 2018; Mo et al., 2015). The slightly positive or negative response in leaf N concentrations to N fertilization across 2012, 2015, and 2017 also revealed that the tropical plants were exposed to a sufficient N supply and that the leaves were failed to absorb excess N (Figures S2 and S3).

In contrast, P addition significantly altered leaf NSC concentrations (both soluble sugars and starch) in the three sampled years. Overall, +P reduced the soluble sugar and starch concentrations by 12.78% and 38.10%, respectively. These results indicate that the NSC dynamics are more sensitive to exogenous P addition rather than to N addition in this forest, which is in agreement with our hypothesis one. Our findings also demonstrate that the tropical trees prefer to lower their starch pools for allocating relatively larger mobile C to enhance plant growth under sufficient P availability (Li, Niu, & Yu, 2016). Additionally, our results reveal that continuous P addition down‐regulated both leaf soluble sugars and starch concentrations of the majority of the species evaluated in this P‐limiting tropical forest (Figure S1). This further supports our hypothesis that the leaf NSCs would be primarily regulated by P availability rather than N availability in this low‐P availability forest. A previous study reported that plant NSC storage was mediated by nutrient availability, with a larger allocation to storage when growth was limited by nutrients (Knox & Clarke, 2005). In this P‐deficient tropical forest, the higher leaf NSC storage was observed in CK plots, and exogenous P addition reduced the allocation of leaf soluble sugars and starch in most of the studied species in the tropical forest, which is consistent with a previous study (Knox & Clarke, 2005). The reduced NSC concentrations may be converted into structural C to form the tissue biomass, which would further enhance plant productivity (Li, Niu, et al., 2016).

### 4.2 Species‐specific effects on leaf NSCs

Leaf soluble sugars, starch, and total NSC (soluble sugars and starch) concentrations generally vary greatly among different plant species (Almeida, Carneiro, Carvalho, Figueiredo‐Ribeiro, & Moraes, 2015; Hoch et al., 2003). The soluble sugar/starch ratios can reflect the NSC allocation pattern in leaves, which is essential for understanding the plant nutrient utilization strategies (Martínez‐Vilalta et al., 2016; Xie et al., 2018). However, the leaf soluble sugars, starch, and NSC concentrations varied greatly across different regions (Druege, Zerche, & Kadner, 2004; Guo et al., 2016; Li, He, et al., 2016; Liu, Su, Li, Lang, & Huang, 2018; Martínez‐Vilalta et al., 2016). In our study, the concentrations of leaf soluble sugar, starch, and NSCs in three of four species were similar to the results from a neighboring tropical forest on Hainan Island (Li, He, et al., 2016), *S. octophylla* was the exception to this pattern, as in this species leaf soluble sugar and starch concentrations were two to four times higher than those of the other three species evaluated in this tropical forest. Plant life history and ecological strategies primarily determine the quantity and allocation of carbohydrates in plants exposed to similar environmental conditions (Hartmann et al., 2018; Newell et al., 2002; Palacio, Maestro, & Montserrat‐Martí, 2007). In this tropical forest, all studied species were understory shade‐tolerant species that well adapted to the shade environment. The C assimilation of these tree species was lower due to their understory shade conditions, while their photosynthetic C assimilation would be primarily limited by continuous nutrient supply status, but not by light availability (Liu et al., 2018; Xie et al., 2018). These findings may provide profound insights into seedling regeneration and ecosystem functions in tropical forests (Santiago et al., 2012).

Leaf expansion may also be closely linked with the storage of NSCs because the long‐distance transport of nutrients could consume a larger amount of energy derived from NSCs (Zhao & Oosterhuis, 2000). Previous studies suggested that a higher concentration of NSCs may be closely related to a larger leaf area (Almeida et al., 2015; Zhao & Oosterhuis, 2000). In 2015, we measured the LMA of these four species included in this study and found that *S. octophylla* had significantly lower LMA than those of *S. bullockii* and *P. rubra* (Mo et al., 2019). Hence, the lower LMA of *S. octophylla* may be attributed to its higher soluble sugars and starch concentrations. In addition, previous studies have suggested that plants with higher initial NSC reserves are more likely to survive when exposed to the adverse environmental conditions (Canham, Kobe, Latty, & Chazdon, 1999; Imaji & Seiwa, 2010). The higher amount of seedling individuals of *S. octophylla* could be explained by the relatively higher leaf NSC storage compared to that of the other species studied in this tropical forest (Table 1).

### 4.3 Leaf NSC and plant adaptation to lower P availability

Previous studies have shown that both N and P addition reduced the leaf NSC (soluble sugar and starch) concentrations in some herbaceous plants, due to the carbohydrate consumption associated with relatively higher plant growth rates under N and P fertilization (Ai et al., 2017; Wang, Xu, et al., 2017). At this study site, we did not observe a significant change in the rate of photosynthesis under N or P addition in these plants (Mo et al., 2019). Nonetheless, we found that P addition significantly decreased the leaf soluble sugar concentrations in our study species. The reduced soluble sugar concentrations may be converted into the leaf tissue, which was explained by the negative correlation between the leaf soluble sugar concentrations and LMA (Table 3). Moreover, the increased structural P fraction further confirmed that P addition enhanced leaf expansion (Mo et al., 2019). Therefore, under P‐insufficient conditions, tropical trees tend to maintain lower P concentrations and higher NSC contents, which may be one potential evolutionary mechanism by which tropical plants have adapted to P‐deficient soil.

Although leaf NSCs are mainly regulated by P availability rather than N availability in this tropical forest, plants may also tend to transform and reallocate NSCs among leaves, branches, stems, and roots to adapt to the adverse environmental conditions. Given that only leaf NSCs were evaluated in this study, the NSC responses to continuous N and P addition and changes in NSC concentrations among different plant organs could be far more complex than the results on leaves reported in this study, more detailed studies are still needed to investigate the leaf NSC responses to N and P availability in a wider range of plant species in tropical forests.

## 5 CONCLUSIONS

In this P‐limited tropical forest, we found that leaf NSCs were primarily regulated by P availability rather than N availability. Our results suggest that the down‐up regulation of leaf soluble sugar and starch concentrations by soil P availability could be a potential mechanism for tropical plants to adapt to low soil P conditions. Since tropical forests are generally limited by P availability globally, a better understanding of plant leaf NSC concentrations and the relationship with soil P availability are of great importance to improve our knowledge on how plants adapt to low‐P soils.

## CONFLICT OF INTERESTS

The authors declared no conflicting interests regarding the publication of this paper.

## AUTHOR CONTRIBUTION

**Qifeng Mo:** Methodology (equal); Writing‐original draft (equal). **Yiqun Chen:** Writing‐review & editing (equal). **Shiqin Yiu:** Writing‐review & editing (equal). **Yingxu Fan:** Writing‐review & editing (equal). **Zhongtong Peng:** Writing‐review & editing (equal). **Wenjuan Wang:** Writing‐review & editing (equal). **Li Zhi'an:** Funding acquisition (equal). **Faming Wang:** Formal analysis (equal); Funding acquisition (lead); Investigation (lead); Methodology (supporting); Project administration (lead); Supervision (equal); Writing‐review & editing (lead).

### Open Research Badges

This article has earned an Open Data Badge for making publicly available the digitally‐shareable data necessary to reproduce the reported results. The data is available at https://doi.org/10.5061/dryad.rbnzs7h8j.

## Supporting information

Appendix S1

## Data Availability

The authors confirm that the data supporting the findings of this study are available within the article and its Appendix S1. All the raw data can be public available in Dryad, Dataset: https://doi.org/10.5061/dryad.rbnzs7h8j.
