# Supplementary material for: Leaf nonstructural carbohydrate concentrations of understory woody species regulated by soil phosphorus availability in a tropical forest
Source: Ecol Evol. 2020 Jul 14;10(15):8429–38. doi: 10.1002/ece3.6549 (PMC7417251; doi:10.1002/ece3.6549)
Supplement: Supplementary file 1 — Appendix S1 [file ECE3-10-8429-s001.docx]

Table S1 Two-way ANOVA of N and P addition on foliar N, P concentrations and N:P ratios, soluble sugar and starch concentration of four woody species in 2012, 2015 and 2017 in a secondary tropical forest

| Year | Species | Variable | N concentration | P concentration | N:P ratio | Soluble sugar concentration | Starch concentration | NSC concentration | Soluble sugar/Starch |
| --- | --- | --- | --- | --- | --- | --- | --- | --- | --- |
| 2012 | *S. bullockii* | N | ***0.040*** | 0.415 | 0.892 | 0.235 | 0.932 | 0.322 | 0.481 |
|  |  | P | ***0.002*** | ***0.041*** | 0.080 | 0.089 | 0.192 | **0.051** | 0.930 |
|  |  | N×P | ***0.000*** | 0.792 | 0.348 | 0.193 | 0.817 | 0.249 | 0.428 |
|  | *U. macrophylla* | N | 0.292 | 0.927 | 0.774 | 0.186 | 0.311 | 0.080 | 0.572 |
|  |  | P | 0.814 | ***0.023*** | ***0.002*** | 0.096 | 0.189 | ***0.029*** | 0.572 |
|  |  | N×P | 0.446 | 0.515 | 0.220 | 0.091 | 0.790 | 0.142 | 0.538 |
|  | *P. rubra* | N | 0.249 | 0.632 | 0.260 | 0.515 | 0.466 | 0.343 | 0.862 |
|  |  | P | 0.900 | ***0.001*** | ***0.000*** | 0.253 | ***0.038*** | ***0.047*** | 0.449 |
|  |  | N×P | 0.545 | 0.716 | 0.455 | 0.548 | 0.300 | 0.299 | 0.828 |
|  | *S. octophylla* | N | 0.785 | 0.899 | 0.940 | 0.769 | ***0.011*** | 0.225 | **0.055** |
|  |  | P | 0.681 | 0.305 | 0.250 | 0.186 | ***0.005*** | ***0.013*** | 0.239 |
|  |  | N×P | ***0.000*** | 0.122 | 0.242 | 0.083 | ***0.046*** | ***0.017*** | 0.827 |
| 2015 | *S. bullockii* | N | ***0.012*** | 0.171 | ***0.004*** | 0.441 | 0.888 | 0.570 | 0.417 |
|  |  | P | 0.328 | ***0.015*** | ***0.000*** | 0.438 | ***0.030*** | 0.308 | ***0.016*** |
|  |  | N×P | 0.598 | 0.494 | 0.609 | 0.943 | 0.256 | 0.429 | 0.551 |
|  | *U. macrophylla* | N | 0.900 | 0.623 | 0.756 | 0.917 | 0.082 | 0.198 | 0.131 |
|  |  | P | 0.266 | ***0.018*** | ***0.040*** | 0.965 | 0.474 | 0.604 | 0.354 |
|  |  | N×P | 0.625 | 0.815 | 0.642 | 0.773 | 0.720 | 0.675 | 0.656 |
|  | *P. rubra* | N | 0.825 | 0.539 | 0.749 | 0.347 | 0.827 | 0.457 | 0.201 |
|  |  | P | 0.900 | ***0.001*** | ***0.000*** | 0.144 | 0.303 | 0.356 | 0.052 |
|  |  | N×P | ***0.016*** | ***0.036*** | 0.374 | 0.410 | 0.104 | 0.193 | 0.307 |
|  | *S. octophylla* | N | 0.837 | 0.285 | 0.290 | 0.740 | 0.377 | 0.661 | 0.836 |
|  |  | P | 0.514 | ***0.015*** | ***0.000*** | ***0.028*** | 0.261 | ***0.003*** | 0.977 |
|  |  | N×P | 0.555 | 0.369 | 0.961 | 0.675 | 0.567 | 0.350 | 0.532 |
| 2017 | *S. bullockii* | N | 0.267 | ***0.027*** | ***0.005*** | 0.367 | 0.921 | 0.442 | 0.404 |
|  |  | P | 0.129 | ***0.004*** | ***0.000*** | 1.000 | 0.190 | 0.661 | 0.511 |
|  |  | N×P | 0.148 | ***0.024*** | ***0.047*** | 0.152 | 0.363 | 0.121 | 0.578 |
|  | *U. macrophylla* | N | ***0.034*** | 0.679 | 0.952 | 0.930 | 0.397 | 0.989 | 0.743 |
|  |  | P | 0.231 | ***0.011*** | ***0.000*** | 0.469 | 0.714 | 0.446 | 0.607 |
|  |  | N×P | 0.146 | 0.401 | 0.163 | 0.310 | 0.626 | 0.339 | 0.228 |
|  | *P. rubra* | N | ***0.000*** | ***0.032*** | 0.624 | 0.522 | 0.730 | 0.505 | 0.634 |
|  |  | P | 0.834 | ***0.000*** | ***0.002*** | ***0.014*** | 0.970 | ***0.049*** | 0.658 |
|  |  | N×P | 0.928 | 0.168 | 0.225 | 0.394 | **0.056** | 0.801 | ***0.015*** |
|  | *S. octophylla* | N | 0.093 | 0.932 | 0.438 | 0.419 | 0.820 | 0.422 | 0.530 |
|  |  | P | 0.251 | 0.267 | 0.549 | 0.506 | 0.737 | 0.449 | 0.595 |
|  |  | N×P | ***0.020*** | 0.668 | 0.192 | 0.127 | 0.099 | 0.061 | 0.402 |

Table S2 Correlation coefficients of leaf N:P stoichiometry and NSC variables of four species in a tropical forest

| Species | Variable | N | P | N:P ratio | Soluble sugar | Starch | NSC | Soluble sugar/Starch |
| --- | --- | --- | --- | --- | --- | --- | --- | --- |
| *S. bullockii* | N | 1.000 |  |  |  |  |  |  |
|  | P | 0.192 | 1.000 |  |  |  |  |  |
|  | N:P ratio | 0.250 | -0.829** | 1.000 |  |  |  |  |
|  | Soluble sugar | 0.396** | -0.027 | 0.239 | 1.000 |  |  |  |
|  | Starch | 0.202 | -0.232 | 0.355** | 0.546** | 1.000 |  |  |
|  | NSC | 0.363** | -0.140 | 0.345** | 0.929** | 0.784** | 1.000 |  |
|  | Soluble sugar/Starch | 0.175 | 0.192 | -0.121 | 0.315* | -0.559** | 0.012 | 1.000 |
| *U. macrophylla* | N | 1.000 |  |  |  |  |  |  |
|  | P | 0.422** | 1.000 |  |  |  |  |  |
|  | N:P ratio | 0.025 | -0.863** | 1.000 |  |  |  |  |
|  | Soluble sugar | 0.055 | 0.294* | -0.270 | 1.000 |  |  |  |
|  | Starch | 0.003 | 0.040 | -0.010 | -0.049 | 1.000 |  |  |
|  | NSC | 0.072 | 0.268 | -0.212 | 0.762** | 0.524** | 1.000 |  |
|  | Soluble sugar/Starch | -0.018 | 0.131 | -0.137 | 0.566** | -0.786** | 0.000 | 1.000 |
| *S. octophylla* | N | 1.000 |  |  |  |  |  |  |
|  | P | 0.293* | 1.000 |  |  |  |  |  |
|  | N:P ratio | 0.453** | -0.604** | 1.000 |  |  |  |  |
|  | Soluble sugar | 0.422** | -0.059 | 0.492** | 1.000 |  |  |  |
|  | Starch | -0.299* | -0.184 | -0.147 | -0.399** | 1.000 |  |  |
|  | NSC | 0.288* | -0.228 | 0.503** | 0.896** | -0.034 | 1.000 |  |
|  | Soluble sugar/Starch | 0.427** | 0.160 | 0.295* | 0.727** | -0.900** | 0.400** | 1.000 |
| *P. rubra* | N | 1.000 |  |  |  |  |  |  |
|  | P | 0.281* | 1.000 |  |  |  |  |  |
|  | N:P ratio | 0.449** | -0.653** | 1.000 |  |  |  |  |
|  | Soluble sugar | 0.147 | -0.183 | 0.320** | 1.000 |  |  |  |
|  | Starch | -0.336** | -0.175 | -0.103 | 0.269* | 1.000 |  |  |
|  | NSC | -0.018 | -0.202 | 0.191 | 0.882** | 0.645** | 1.000 |  |
|  | Soluble sugar/Starch | 0.416** | 0.028 | 0.312** | 0.350** | -0.752** | -0.046 | 1.000 |

Data are Spearman correlation coefficients. ** indicates an extremely significant correlation at the 0.01 level (bilateral), * indicates a significant correlation at the 0.05 level (bilateral).

Table S3 Species-based correlation coefficients of leaf N:P stoichiometry and NSC variables of four species in a tropical forest in 2015, where NSC is non-structural carbohydrates, LMA is leaf mass per unit area; [N] is N concentrations, [P] is P concentrations, PNUE is photosynthetic N-use efficiency and PPUE is photosynthetic P-use efficiency

| Species | Variables | Soluble sugar | Starch | NSC | Soluble sugar/ Starch |
| --- | --- | --- | --- | --- | --- |
| *Syzygium bullockii* | Photosynthesis | 0.118 | 0.108 | 0.150 | 0.041 |
|  | Leaf area | 0.395 | -0.026 | 0.219 | 0.177 |
|  | LMA | -0.278 | 0.083 | -0.107 | -0.266 |
|  | [N] | -0.019 | -0.231 | -0.180 | 0.265 |
|  | [P] | 0.327 | -0.211 | 0.042 | 0.251 |
|  | N:P ratios | -0.158 | 0.195 | 0.048 | -0.195 |
|  | PNUE | 0.091 | 0.118 | 0.141 | 0.005 |
|  | PPUE | 0.022 | 0.180 | 0.145 | -0.046 |
|  | Structural P | 0.347 | -0.100 | 0.136 | 0.149 |
|  | Metabolic P | 0.288 | -0.243 | -0.004 | 0.275 |
|  | Nucleic acid P | 0.156 | -0.160 | -0.023 | 0.169 |
|  | Residual P | 0.017 | 0.017 | 0.023 | -0.002 |
| *Uvaria microcarpa* | Photosynthesis | -0.378 | 0.211 | -0.011 | -0.382 |
|  | Leaf area | 0.171 | -0.348 | -0.198 | 0.331 |
|  | LMA | **0.549*** | -0.160 | 0.134 | 0.421 |
|  | [N] | 0.029 | 0.090 | 0.086 | 0.017 |
|  | [P] | 0.093 | 0.069 | 0.100 | 0.048 |
|  | N:P ratios | 0.013 | 0.008 | 0.013 | -0.064 |
|  | PNUE | -0.406 | 0.186 | -0.044 | -0.399 |
|  | PPUE | -0.352 | 0.095 | -0.092 | -0.349 |
|  | Structural P | 0.382 | 0.217 | 0.356 | 0.051 |
|  | Metabolic P | 0.073 | 0.079 | 0.098 | 0.018 |
|  | Nucleic acid P | -0.017 | -0.159 | -0.136 | 0.176 |
|  | Residual P | -0.318 | -0.028 | -0.174 | -0.015 |
| *Psychotria rubra* | Photosynthesis | 0.138 | 0.272 | 0.316 | 0.048 |
|  | Leaf area | 0.298 | -0.305 | 0.057 | 0.277 |
|  | LMA | **0.479*** | 0.049 | **0.463*** | -0.043 |
|  | [N] | 0.170 | -0.017 | 0.141 | 0.059 |
|  | [P] | -0.418 | -0.132 | **-0.486*** | -0.077 |
|  | N:P ratios | **0.487*** | 0.329 | **0.662*** | -0.025 |
|  | PNUE | 0.140 | 0.223 | 0.284 | 0.099 |
|  | PPUE | 0.295 | 0.404 | **0.542*** | 0.002 |
|  | Structural P | -0.247 | -0.059 | -0.262 | -0.036 |
|  | Metabolic P | -0.426 | -0.134 | **-0.474*** | -0.104 |
|  | Nucleic acid P | **-0.470*** | -0.259 | **-0.599*** | 0.052 |
|  | Residual P | -0.180 | 0.123 | -0.077 | -0.150 |
| *Schefflera octophyll* | Photosynthesis | **-0.440*** | -0.025 | **-0.399*** | -0.288 |
|  | Leaf area | -0.167 | 0.123 | -0.102 | -0.183 |
|  | LMA | 0.106 | 0.242 | 0.181 | -0.036 |
|  | [N] | -0.046 | 0.049 | -0.022 | -0.084 |
|  | [P] | 0.338 | 0.224 | 0.378 | 0.002 |
|  | N:P ratios | -0.319 | -0.218 | -0.360 | -0.003 |
|  | PNUE | -0.386 | -0.018 | -0.349 | -0.256 |
|  | PPUE | **-0.452*** | -0.007 | **-0.399*** | -0.296 |
|  | Structural P | **0.424*** | -0.037 | 0.359 | 0.308 |
|  | Metabolic P | 0.296 | 0.221 | 0.341 | -0.025 |
|  | Nucleic acid P | 0.347 | 0.118 | 0.348 | 0.057 |
|  | Residual P | 0.099 | 0.303 | 0.197 | -0.196 |

Table S4 Treatment-based correlation coefficients of leaf N:P stoichiometry and NSC variables of four species in a tropical forest in 2015, where NSC is non-structural carbohydrates, LMA is leaf mass per unit area; [N] is N concentrations, [P] is P concentrations, PNUE is photosynthetic N-use efficiency and PPUE is photosynthetic P-use efficiency

| Treatments | Variables | Soluble sugar | Starch | NSC | Soluble sugar/ Starch |
| --- | --- | --- | --- | --- | --- |
| CK | Photosynthesis | 0.253 | 0.389 | 0.336 | 0.163 |
|  | Leaf area | **0.496*** | 0.021 | **0.448*** | **0.518*** |
|  | LMA | -0.335 | **-0.436*** | -0.423 | -0.058 |
|  | [N] | 0.395 | 0.056 | 0.367 | 0.268 |
|  | [P] | 0.071 | -0.086 | 0.038 | 0.085 |
|  | N:P ratios | **0.449*** | 0.174 | **0.449*** | 0.255 |
|  | PNUE | 0.099 | 0.298 | 0.173 | 0.090 |
|  | PPUE | 0.257 | 0.403 | 0.344 | 0.163 |
|  | Structural P | **0.662**** | 0.249 | **0.660**** | **0.480*** |
|  | Metabolic P | 0.150 | 0.033 | 0.143 | 0.152 |
|  | Nucleic acid P | **-0.537**** | -0.211 | **-0.539**** | -0.307 |
|  | Residual P | **-0.462*** | -0.278 | **-0.491*** | -0.363 |
| +N | Photosynthesis | 0.165 | -0.088 | 0.143 | 0.218 |
|  | Leaf area | **0.619**** | 0.268 | **0.644**** | 0.439 |
|  | LMA | -0.257 | 0.188 | -0.213 | -0.190 |
|  | [N] | 0.261 | 0.074 | 0.265 | 0.222 |
|  | [P] | 0.235 | 0.241 | 0.270 | 0.117 |
|  | N:P ratios | -0.063 | -0.343 | -0.123 | 0.120 |
|  | PNUE | 0.044 | -0.192 | 0.007 | 0.161 |
|  | PPUE | 0.015 | -0.278 | -0.036 | 0.180 |
|  | Structural P | **0.632**** | **0.555**** | **0.710**** | 0.260 |
|  | Metabolic P | 0.281 | 0.337 | 0.331 | 0.098 |
|  | Nucleic acid P | -0.229 | 0.174 | -0.188 | -0.295 |
|  | Residual P | -0.212 | -0.340 | -0.266 | 0.038 |
| +P | Photosynthesis | -0.234 | 0.045 | -0.201 | -0.292 |
|  | Leaf area | **0.614*** | 0.330 | **0.611**** | **0.420*** |
|  | LMA | **-0.610**** | -0.282 | **-0.599**** | **-0.492*** |
|  | [N] | 0.142 | 0.254 | 0.175 | 0.019 |
|  | [P] | 0.001 | -0.048 | -0.009 | -0.002 |
|  | N:P ratios | -0.007 | 0.181 | 0.028 | -0.069 |
|  | PNUE | -0.270 | -0.101 | -0.261 | -0.239 |
|  | PPUE | -0.191 | 0.094 | -0.153 | -0.267 |
|  | Structural P | **0.525*** | 0.162 | **0.500*** | 0.406 |
|  | Metabolic P | -0.001 | -0.048 | -0.010 | 0.001 |
|  | Nucleic acid P | -0.232 | -0.329 | -0.270 | -0.057 |
|  | Residual P | **-0.415*** | -0.059 | -0.383 | -0.381 |
| +NP | Photosynthesis | 0.097 | 0.126 | 0.123 | 0.110 |
|  | Leaf area | **0.434*** | 0.118 | **0.439*** | 0.194 |
|  | LMA | **-0.697**** | 0.061 | **-0.642**** | **-0.745**** |
|  | [N] | 0.158 | 0.421 | 0.255 | -0.228 |
|  | [P] | 0.276 | 0.396 | 0.361 | 0.044 |
|  | N:P ratios | -0.222 | -0.195 | -0.258 | -0.222 |
|  | PNUE | 0.040 | 0.022 | 0.043 | 0.146 |
|  | PPUE | -0.179 | 0.041 | -0.159 | -0.114 |
|  | Structural P | 0.388 | 0.237 | 0.426 | 0.251 |
|  | Metabolic P | 0.265 | 0.331 | 0.334 | 0.068 |
|  | Nucleic acid P | 0.100 | 0.194 | 0.144 | 0.091 |
|  | Residual P | -0.231 | 0.156 | -0.179 | -0.364 |

Table S5 Significance (*p*-values) of terms from linear mixed model analysis for foliar N, P concentrations and N:P ratios of four woody species in a tropical forest following the fertilization with nitrogen (N) and phosphorus (P), where S is species; N is N-addition; and P is P-addition.

| Variable | N concentration | P concentration | N:P ratio |
| --- | --- | --- | --- |
| S | ***0.000*** | 0.623 | ***0.010*** |
| N | ***0.000*** | 0.178 | 0.104 |
| P | 0.147 | ***0.000*** | ***0.000*** |
| S×N | ***0.005*** | ***0.014*** | 0.252 |
| S×P | 0.728 | 0.146 | 0.094 |
| N×P | 0.648 | 0.056 | 0.381 |
| S×N×P | ***0.025*** | 0.199 | 0.058 |

Fig. S1 Effects of N and P addition on foliar N, P concentrations, N:P ratios of four woody species sampled in 2012, 2015 and 2017 in a secondary tropical forest of southern China. Error bars were standard deviations. The lowercase indicated the significant difference among four treatments with each species and each year. The N, P and N:P ratios of the studied four species in 2012, 2015 and 2017 was cited in the previous paper ([Mo et al., 2015](#_ENREF_26)a, 2015b, 2019).

Fig. S2 Relative effects (RE) of leaf N concentrations (a, b, c), P concentrations (d, e, f) and N:P ratios (g, h, i) under +N, +P, and +NP treatments of four woody tree species in a tropical forest of southern China. Error bars were standard deviations. RE was quantified by the ratio of the variable in the experimental group (+N, +P, +NP) to the control group (CK) minus one. The N, P and N:P ratios of the studied four species in 2012, 2015 and 2017 was cited in the previous paper ([Mo et al., 2015](#_ENREF_26)a; 2015b, 2019).

Fig. S3 Foliar sucrose and fructose concentrations of four woody species sampled in 2012, 2015 and 2017 in a secondary tropical forest of southern China. The lowercase indicated the significant difference among four treatments with each species and each year.
